# “Scapegoat” for Offline Consumption: Online Review Response to Social Exclusion

**DOI:** 10.3389/fpsyg.2021.783483

**Published:** 2021-12-02

**Authors:** Shichang Liang, Yuxuan Chu, Yunshan Wang, Ziqi Zhang, Yunjie Wu, Yaping Chang

**Affiliations:** ^1^School of Business Administration, Guangxi University, Nanning, China; ^2^Third Department of Architecture, Hualan Design (Group) Co., Ltd., Nanning, China; ^3^Renmin Business School, Renmin University of China, Beijing, China; ^4^School of Business, Sun Yat-sen University, Guangzhou, China; ^5^School of Management, Huazhong University of Science and Technology, Wuhan, China

**Keywords:** social exclusion, online review, self-protection, self-serving bias, brand awareness

## Abstract

Previous research has mostly focused on Internet use behaviors, such as usage time of the Internet or social media after individuals experienced offline social exclusion. However, the extant literature has ignored online response behaviors, such as online review responses to social exclusion. To address this gap, drawing on self-protection and self-serving bias, we proposed three hypotheses that examine the effect of offline social exclusion on online reviews, which are verified by two studies using different simulating scenarios with 464 participants. The results show that when individuals are socially excluded offline, regardless of where the exclusion comes from (businesses or peers), they will be more likely to give negative online reviews. In addition, brand awareness moderates the effect of offline social exclusion on online reviews. Specifically, if the brand is less known, compared with social inclusion, offline social exclusion will lead individuals to give more negative online reviews; conversely, for well-known brands, no significant difference exists in the online reviews between social exclusion and inclusion.

## Introduction

With the rapid development of online shopping, an increasing number of individuals have been using online platforms to book restaurants, movies, and ball games, among others. After their offline consumption, individuals will give their reviews on such online reservation platforms. These online reviews play an extremely important role in the attitude of customers, which indicates that merchants value these reviews considerably. Online reviews are important for businesses, especially how to create a good offline consumption experience to avoid negative online reviews, which has become a great problem faced by merchants. However, the content of experience of individuals during offline consumption is highly diverse, especially when it comes to contact with groups or individuals in the consumption scene, which is often full of uncertainty. For example, a man booked a long-awaited steak set meal online to celebrate his birthday alone. When he arrived at this restaurant on time, he came across his best friends who were also having a party here, and immediately he felt he has been excluded by his friends. In this case, although the steak meal of this restaurant suited his taste well, he still gave a negative review to his dinner booking on the platform just for being excluded. The restaurant may have nothing to do with the situation of the man, but he has already left a negative review of the restaurant on the platform. Can merchants avoid such an accident, being scapegoats for the unfortunate experience of customers that is irrelevant to the shops? This study addresses this issue by examining the effect of offline social exclusion on online reviews.

Many researchers have examined the influence of offline social exclusion on online behaviors. For example, social exclusion results in frequent use of the Internet by individuals (Lin et al., 2017). In addition, in comparison with those who have not experienced social exclusion, individuals who have experienced it prefer to use social networking sites (SNS; Lin et al., 2017), communicate online with a stranger of the opposite sex (vs. playing online games; Gross, 2006), use new media (i.e., computational media communication; Tom Tong and Walther, 2011), use social media to seek associations (Ahn and Shin, 2013), and seek revenge on the site (Chen, 2012). The extant research has mostly focused on Internet use behaviors, such as using the Internet or online social media after individuals experienced offline social exclusion, that is, the effect of social exclusion on online behaviors. However, these studies have not discussed what happens to online reviews after encountering social exclusion. To address this gap, this study examines how offline social exclusion influences online reviews.

On the basis of self-protection and self-serving bias theories (Miller and Ross, 1975; Mezulis et al., 2004) and attribution theory (Kelley, 1967), this study proposes that offline social exclusion has a significant negative influence on online reviews of individuals. Given that individuals often attribute unfortunate events to the situation rather than to themselves, they tend to attribute to consumption scenarios once they encounter social exclusion, which leads to negative reviews to merchants on online platforms. In addition, given that brand awareness can affect the perception of quality of consumers (Lu et al., 2014), it also moderates the effect of offline social exclusion on online reviews. Specifically, when a shop brand has a low profile, individuals will judge by what they experience during offline consumption, which indicates that offline scenarios lead has a positive effect on reviews. Thus, when individuals feel that they are socially excluded during offline consumption, they will attribute it to the offline scenario and then give negative reviews. By contrast, when a shop brand has a high profile, as perceiving high quality, individuals attribute negative emotions to themselves rather than the shop. That is, regardless of whether individuals encounter social exclusion, they will not give negative online reviews.

This article is structured as follows. The first section introduces the theoretical background and research hypotheses. The second and third sections present Studies 1 and 2, which examine the main effect and the moderated effect of brand awareness, respectively. The fourth section includes the conclusion, the theoretical and managerial implications, and research limitations.

## Theoretical Background and Research Hypotheses

### Social Exclusion and Online Behavior

Social exclusion refers to a state of being isolated or excluded from other individuals or groups (Williams et al., 2000). Many studies have investigated the effect of social exclusion on consumer behavior. For example, in comparison with ambiguous rejection, explicit rejection can lead to less emotional toil (Freedman et al., 2016). When consumers encounter social exclusion, they tend to prefer nostalgic products (Loveland et al., 2010; Xu and Jin, 2020) and purchase products strategically, such as buying products that symbolize membership of a group (Mead et al., 2011) and ones with more visual density (Su et al., 2019). When people feel that they are socially excluded, they prefer to select the brand that will help them become similar to the group (Dommer et al., 2013), some unique products (Echo Wen et al., 2014), green products (Guo et al., 2020), and retails with crowded space to purchase (Thomas and Saenger, 2020). Furthermore, they tend to pursue financial opportunities with greater risks that earn more potential profits (Duclos et al., 2013), more conspicuous consumption behaviors (Liang et al., 2018), and more transfer behaviors (Su et al., 2017) when they are in a social exclusion condition. In addition, social exclusion can influence females more than males on negative attitudes and the sense of control of merchants (Hwang and Mattila, 2019).

Social exclusion has been proven to decrease prosocial behaviors, such as less inclination to help or cooperate with them (Twenge et al., 2007), which prevents them from sharing the experience to others. However, when it comes to the Internet, offline social exclusion can affect the online behaviors of an individual (Lu and Sinha, 2017). For example, in comparison with those who have not experienced social exclusion, individuals who experience it prefer to use SNS to share this terrible experience (Lin et al., 2017). These people are more likely to communicate online with a stranger of the opposite sex (vs. playing online games; Gross, 2006) or use new social media (i.e., computational media communication; Tom Tong and Walther, 2011; Mourey et al., 2017) to seek associations (Ahn and Shin, 2013). In order to release this kind of pressure, they will seek revenge on the site (Chen, 2012), recover the threat of ownership through the use of SNS such as Facebook (Knausenberger and Echterhoff, 2018), or ease their mood when communicating with an online chatbot (de Gennaro et al., 2019). Some scholars also found that offline social exclusion could lead to present prosocial behaviors (Lutz and Schneider, 2020) and spend longer time on font colors in SNS (Lee and Chiou, 2013). These extant studies have indicated that social exclusion affects Internet use behavior. However, they do not consider the influence of offline social exclusion on online response behavior, such as online reviews. To address this gap, this study discusses the effect of offline social exclusion on online reviews.

### Antecedent Variables of Online Reviews

Consumers often search the reviews on online platforms because they believe that most of the reviews are credible (Chen and Xie, 2008; Allard et al., 2020). Online reviews refer to the feedback that reflects the feeling of an online purchase experience, which is divided into negative and positive ways. Positive online reviews lead to more sales through enhancing the positive attitude and expectation of this company, whereas negative online reviews can decrease the evaluations and purchase intention (Ho-Dac et al., 2013; Chevalier and Mayzlin, 2018; Liu, 2018; Guerreiro and Rita, 2020). In addition, some scholars have proposed the boundary conditions of the effects of negative and positive online reviews on product choice. For example, Reich and Maglio (2019) found that positive online reviews that contain a fail purchase experience in another place can be more persuasive than common positive reviews. Rocklage and Fazio (2020) proposed that positive online reviews can arouse positive emotions when it comes to hedonic products rather than functional products. In addition, Berger et al. (2010) found that negative online reviews can enhance the short-term sales of an unknown brand because these reviews help increase the awareness of this brand. Allard et al. (2020) found that unfair negative online reviews (i.e., a negative online review that does not reflect the real condition) help arouse the empathy and then the positive response of consumers. Therefore, the extant research has focused on the moderated effect of the two types of online review on the attitudes and choices of consumers.

Some scholars have examined the motives for consumers to read or write online reviews. Hennig-Thurau et al. (2004) investigated the motives of writing online reviews from the perspectives of focus-related utility, consumption utility, and approval utility, which present the eagerness to interact with others, pursue economic rewards, and help other consumers. Ghazi (2017) found that the motives for writing positive and negative online reviews are extremely different. For positive reviews, consumers are willing to write online reviews that can help the company and other consumers, whereas for negative reviews, consumers tend to vent their negative emotions and some warnings through online reviews. On the basis of incentive programs for online reviewers, Liu et al. (2019) proposed that those who have already written several reviews will pay more effort to pursue the reward from the company, and a large number of online reviews will eager others to participate in this process. Moses et al. (2018) examined the effect of different self-construal on participating in online reviews and designed an interactive online feature to figure out the social motives for writing reviews. Dixit et al. (2019) proposed that subjective norms and behavior control can be used to predict the motive of writing online reviews. However, to the best of our knowledge, no research has taken offline social exclusion as the antecedent variable on online reviews, although offline social exclusion is an overly common scene in the offline consumption environment when individuals use online platforms to book something and consume offline.

### Social Exclusion and Online Reviews

Attribution theory proposes that people will attribute their unfortunate experience to the external environment or themselves (Kelley, 1967). Styles of individual attribution depend on internality, that is, attributing to internal or external factors (Hymel et al., 1985; Newall et al., 2009). In contrast, according to self-protection or self-serving bias theory, most people tend to attribute success to themselves and failure to the situation (i.e., denial of self-responsibility; Miller and Ross, 1975; Mezulis et al., 2004) because people are willing to protect their self-concept positively (Campbell and Sedikides, 1999), which is defined as those who have different stable and definite characters in the phenomenal field (Syngg and Combs, 1949). Especially, people who pursue a goal independently (Sedikides et al., 1998) and feel more self-threat (i.e., a feeling that is generated by questioning, challenging, or mocking favorable ideas) will generate self-serving bias and self-protection. That is, more self-threat feeling can lead to more self-serving bias and self-protection (Campbell and Sedikides, 1999). In addition, social exclusion has been proved to result in less prosocial behavior (Twenge et al., 2007; Kiat et al., 2018) and impaired cognitive functioning (Baumeister et al., 2002), which is the same as self-threat (Gaspar, 2012; Thomas and Saenger, 2020). Therefore, on the scene of offline–online consumption, once consumers encounter offline social exclusion in a consumption situation, given that individuals usually attribute unfortunate events to the situation rather than to themselves, they tend to attribute the negative result to the consumption scenario (i.e., the shop), which will result in dissatisfaction with the consumption and finally more likely to give negative online reviews to the shop than in social inclusion. By contrast, if consumers come across social inclusion during consumption, then they will be less sensitive and responsive to consumption scenarios due to the lack of self-service bias or self-protection awareness than in social exclusion. Therefore, consumers generally do not give negative online reviews when they experience social inclusion.

On the basis of the above reasoning, the following hypotheses are formulated:

*H1*: Consumers are more likely to give negative online reviews to the shop when they are in offline social exclusion conditions than in social inclusion conditions.

*H2*: Situational attribution mediates the influence of social exclusion on online reviews.

### Brand Awareness

Brand awareness represents the influence of merchants among groups of consumers. In other words, brand awareness equals the recognition level of a brand name based on perceptual frequency, regardless of product categories (Hellofs and Jacobson, 1999). For example, among brands of sport equipment, Little Sheep and Haidilao Hot Pot (two Chinese brands) are widely recognized for high quality and satisfaction in service and products. However, in terms of service satisfaction, Haidilao Hot Pot is more widely recognized and has a greater brand reputation than Little Sheep, which implies that its brand awareness is higher. Moreover, this difference in brand awareness will affect the perceptions and behavior of consumers (Oh, 2000). Therefore, brand awareness may affect the relationship between offline social exclusion and online reviews.

The influence of brand awareness on consumers depends on the perception of brand quality. The investment of brand awareness is expected to produce reward, but a brand with low quality will decrease the benefit of this investment; thus, high-quality brand has dynamics and can afford to improve and finally reach high brand awareness (Kirmani and Rao, 2000; Erdem et al., 2018). A well-known brand is most likely to attract the attention of consumers and is likely to be perceived as high quality (Dodds and Monroe, 1985; Teas and Agarwal, 1997). In addition, when consumers are not familiar with the products, they tend to purchase products of well-known brands because well-known brands make consumers think that the brand is popular and can satiate their psychological demands (Hellofs and Jacobson, 1999). On online behavior of booking consumption, consumers tend to pay attention to the brand awareness of consuming service or products and judge the popularity of the merchant by using brand awareness. When a shop brand has a high profile, as perceiving the high quality of the service or products and great popularity of the shop, customers attribute negative emotions to themselves rather than the shop. Therefore, when the shop brand is well known, consumers are less likely to give negative online reviews regardless whether they encounter social exclusion.

On the contrary, if the shop brand awareness is less known, then the trust of consumers toward the merchants will be extremely low because they have little knowledge of these brands (Lu et al., 2014), and they tend to attribute the low brand awareness to the low quality of products and services provided (Kirmani and Rao, 2000). Therefore, consumers tend to have more self-protection and self-serving bias consciousness with the merchant of a less-known brand than of a well-known brand. Thus, if any problem occurs during offline consumption, even when it is caused by the consumers, then the merchants will be the most to be blamed. Therefore, when consumers encounter social exclusion during offline consumption of online booking products or services with low brand awareness, they will blame it on the merchants and give negative online reviews, no matter if the exclusion comes from their peers or consumption experience.

*H3*: Brand awareness moderates the effect of social exclusion on online reviews.

Specifically, when the shop brand is less known, consumers who are in offline social exclusion condition will give more negative online reviews to the shop than in a social inclusion condition. Nevertheless, when the shop brand is well known, no significant influence exists on online reviews between social exclusion and social inclusion.

**FIGURE 1:**
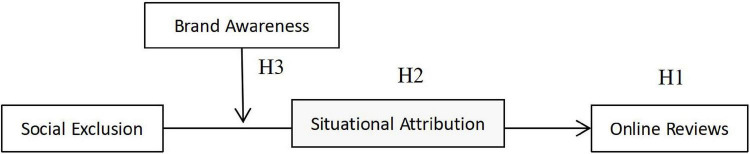
Framework of research hypotheses.

The theoretical framework of this study is presented in Figure 1.

## Study 1

The purpose of Study 1 is to verify the negative effect of offline social exclusion on online reviews (i.e., H1) and the mediating effect of situation attribution in this effect (i.e., H2). Study 1 uses the way of scenario simulation to manipulate offline social exclusion; specifically, individuals are rejected by peers or waiters in the restaurant.

### Methods

A total of 166 undergraduates (93 females) were recruited and given extra course credit for their participation. The average age of the participants was 21 years (*SD* = 1.34). The participants were randomly assigned to a 2 (implementer of social exclusion: peer vs. waiter) × 2 (social exclusion: exclusion vs. inclusion) between-subjects design.

When the participants arrived at the experimental site, they were informed to read the materials of offline consumption experience (see Supplementary Appendix 1) and were then required to complete a survey aiming at improving the online booking platform service. To increase the credibility of the survey and achieve better experimental results, the participants were told that the survey was launched by a famous online booking platform in cooperation with a professor, aiming at collecting opinions about their online platform. After reading the materials of the offline consumption experience, the participants were asked to imagine that they were really in that scene where the reading materials show. Then, they were asked to complete a paper-and-pencil questionnaire about the scene and online platform.

The participants were initially asked to rate the level of being rejected in the offline consumption scenario (1 = very low, 7 = very high). Then, the participants were told to rate four items related to the restaurant, which include “how you feel with the taste of the reserved restaurant?” “how you feel with the environment of the reserved restaurant?” “how you feel with the service of the reserved restaurant?” (1 = very dissatisfied, 7 = very satisfied), and “what is your assessment to the restaurant you booked?” (1–7 stars); the scores of these items were averaged to the evaluation of the restaurant. In addition, the participants were asked to complete the scale of situational attribution. That is “how much the situation affects you in the restaurant scene?” (1 = very low, 7 = very high).

### Data Analysis and Results

#### Manipulation Check

As expected, in comparison with being accepted by the peer or waiter, the participants felt more rejected when they were rejected [*M*_*exclusion*_ = 4.76, *SD* = 1.28 vs. *M*_*inclusion*_ = 3.80, *SD* = 1.43, *F*(1,188) = 23.78, *p* = 0.00, Cohen’s *d* = 0.707]. Thus, the manipulation of offline social exclusion was successful.

#### Hypothesis Testing

The results of ANOVA analysis of online review (Cronbach’s alpha = 0.89) revealed that the main effect of offline social exclusion on online reviews [*F*(1,186) = 63.46, *p* = 0.000] was significant, which indicated that there existed significant differences in online reviews of restaurants given by individuals who have encountered social exclusion. Moreover, the implementer of social exclusion had no significant main effect on online reviews [*F*(1,186) = 1.54, *p* = 0.217]. In addition, the interaction terms of social exclusion and the implementer had no significant effect [*F*(1,186) = 0.03, *p* = 0.853], indicating that the interaction between social exclusion and the implementer of social exclusion did not affect online reviews (see Table 1).

**TABLE 1:** ANOVA analysis of study 1.

Variable	SS	df	MS	*F*	*p*
Social exclusion	59.69	1	59.69	63.46	0.000
Implementer	1.45	1	1.45	1.54	0.217
Social exclusion × Implementer	0.03	1	0.03	0.03	0.853

**FIGURE 2:**
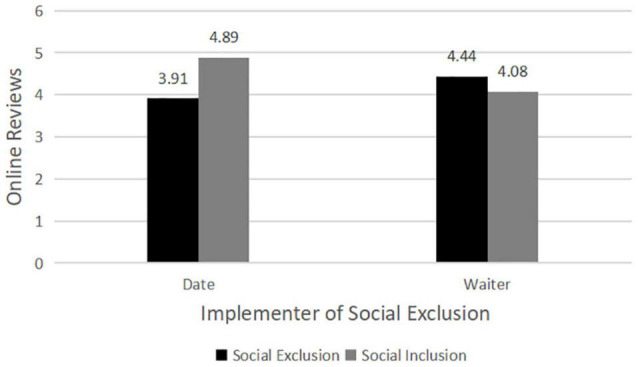
The impact of social exclusion on online reviews (study 1).

The results of further analysis showed that the participants in dating failure scenario (social exclusion from date) gave more negative online reviews to the restaurant compared with dating success scenario [social inclusion from date; *M*_*exclusion*_ = 4.03, *SD* = 1.25 vs. *M*_*inclusion*_ = 5.12, *SD* = 0.70, *F*(1,91) = 27.21, *p* = 0.000, Cohen’s *d* = 1.076]. Likewise, the participants in the service failure scenario (social exclusion from waiter) tended to give more negative online reviews to the restaurant compared with service success scenario [social inclusion from waiter; *M*_*exclusion*_ = 3.83, *SD* = 1.04 vs. *M*_*inclusion*_ = 4.97, *SD* = 0.80, *F*(1,95) = 37.13, *p* = 0.000, Cohen’s *d* = 1.229]. As a result, H1 was supported (see Figure 2).

The results of ANOVA analysis showed that the main effect of offline social exclusion on situational attribution was significant [*M*_*exclusion*_ = 5.05, *SD* = 1.50 vs. *M*_*inclusion*_ = 3.06, *SD* = 1.37, *F*(1,188) = 90.55, *p* < 0.01, Cohen’s *d* = 1.385]. In other words, in comparison with social inclusion, when the participants were socially excluded, they were more likely to attribute to the situation. By using Model 4 as suggested by Hayes (2013), a bootstrapping analysis found that situational attribution mediated the effect of offline social exclusion on online reviews, indirect effect on online reviews through situational attribution [*B* = 0.909, 95% confidence interval (CI): (0.667, 1.196), not including zero], and a direct effect on online reviews that was not through situational attribution [*B* = 0.214, 95% CI: (−0.463, 0.034), includes zero]. This result showed that situational attribution mediated the effect of offline social exclusion on online reviews. Therefore, H2 was supported.

### Discussion

The results of Study 1 showed that in comparison with social inclusion, the participants were more likely give negative reviews to the restaurant when they encountered social exclusion, whether it came from their peer or the waiter (H1), which also indicated that personal experience and service quality considerably affected the online reviews. Study 1 also verified that situational attribution mediated the effect of offline social exclusion on online reviews (H2).

## Study 2

Study 2 aims to verify that brand awareness moderates the effect of offline social exclusion on online reviews (H3). Specifically, when the shop is less known, consumers who are in an offline social exclusion condition will more likely give negative online reviews to the shop than in a social inclusion condition. However, when the shop is well known, no significant effect exists on online reviews between social exclusion and social inclusion. To achieve this goal, we use the same process with different scenes in Study 1 and add the manipulation of brand awareness into the scenario.

### Methods

A total of 218 undergraduates (122 females) were recruited and given monetary compensation for their participation. The average age of the participants was 21 years (*SD* = 1.34). The participants were randomly assigned to a 2 (brand awareness: less known vs. well known) × 2 (social exclusion: exclusion vs. inclusion) between-subjects design.

By using the same process in Study 1, when the participants arrived, they were informed to read the materials of offline consumption experience (see Supplementary Appendix 2) and then need to complete a survey. Different from Study 1, Study 2 used hotpot restaurants by using two restaurant names, namely, Haidilao (well known as a hotpot restaurant in China) and Xiaofuzi (unknown hotpot restaurant). Then, the participants were asked to complete the same questionnaire used in Study 1, except an additional item used to check the manipulation of brand awareness was included, that is, “What do you think of the reputation of the restaurant?” (1 = is very low, 7 = very high).

### Data Analysis and Results

#### Manipulation Check

As expected, the response of the participants to the brand awareness of the hotpot restaurant of Haidilao was higher than Xiaofuzi [*M*_*Haidilao*_ = 5.18, *SD* = 1.17 vs. *M*_*Xiaofuzi*_ = 3.07, *SD* = 1.62, *F*(1,216) = 120.23, *p* < 0.01, Cohen’s *d* = 1.493]. In comparison with social inclusion, the participants felt more exclusive when they were in social exclusion [*M*_*exclusion*_ = 4.81, *SD* = 1.53 vs. *M*_*inclusion*_ = 3.48, *SD* = 1.71, *F*(1,216) = 36.40, *p* < 0.01, Cohen’s *d* = 0.82], which confirmed the success of the experimental manipulation scenarios.

#### Hypothesis Testing

We conducted an ANOVA with offline social exclusion and brand awareness as independent variables and the online reviews (Cronbach’s alpha = 0.88) as the dependent variable. The results revealed that there existed a main effect of social exclusion on online reviews [*F*(1,214) = 5.42, *p* < 0.05]. In addition, no main significant effect of brand awareness was observed on online reviews [*F*(1,214) = 1.08, *p* > 0.3]. Most importantly, a significant interactive effect of social exclusion and brand awareness existed on online reviews [*F*(1,214) = 24.07, *p* < 0.01; see Table 2].

**TABLE 2:** ANOVA analysis of study 2.

Variable	SS	df	MS	*F*	*p*
Social exclusion	5.491	1	5.491	5.421	0.021
Brand awareness	1.093	1	1.093	1.079	0.300
Social exclusion × Brand awareness	24.379	1	24.379	24.067	0.000

**FIGURE 3:**
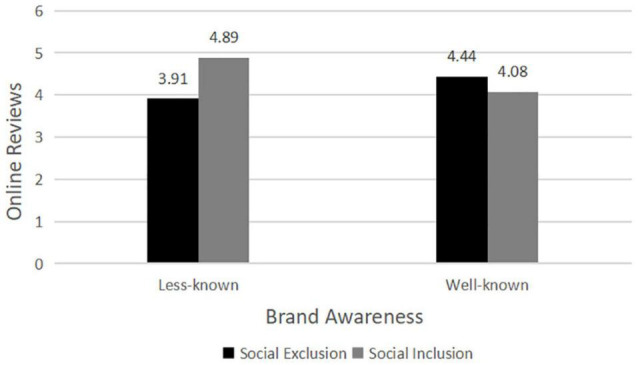
The impact of brand awareness and social exclusion on online reviews.

The results of further analysis showed that the participants would more likely give negative online reviews to the restaurant encountering social exclusion (vs. social inclusion) when the restaurant is less known [*M*_*exclusion*_ = 3.91, *SD* = 1.13 vs. *M*_*inclusion*_ = 4.89, *SD* = 0.91, *F*(1,108) = 25.37, *p* < 0.01, Cohen’s *d* = 0.955]. In contrast, when the restaurant was well known, no significant difference in online reviews [*M*_*exclusion*_ = 4.44, *SD* = 0.93 vs. *M*_*inclusion*_ = 4.08, *SD* = 1.04, *F*(1,106) = 3.44, *p* > 0.05, Cohen’s *d* = 0.365] was observed between social exclusion and social inclusion. Therefore, H3 was supported (see Figure 3).

### Discussion

Using the same process of Study 1, Study 2 verified that brand awareness moderated the effect of offline social exclusion on online reviews using the restaurant scene. Specifically, if the restaurant is less known (Xiaofuzi), then, the participants who were socially excluded were inclined to give the restaurant more negative online reviews compared with being socially inclusive. In contrast, if the restaurant is well known (Haidilao), no significant difference was observed in online reviews of the restaurant between social exclusion and social inclusion. These results showed that brand awareness plays an important role in the effect of social exclusion on online reviews.

## General Discussion

### Conclusion

At present, people are increasingly relying on online platforms or social media to make online reservations before offline consumption, such as restaurants, movies, hotels, and tours. Then, after offline consumption, consumers will post reviews on the online platform as feedback of the experience. With the development of online shopping, more and more consumers prefer to book online before offline consumption. The effect of offline consumption on online reviews has also attracted more attention of researchers. By simulating offline dating consumption scenarios, this study examines the influence of offline social exclusion on online reviews. By using two experiments, this work finds that consumers who experience offline social exclusion will more likely give negative online reviews to the shop than in a social inclusion condition. In addition, brand awareness moderates the effect of social exclusion on online reviews. Specifically, if the brand of the shop is less known, customers tend to give more negative reviews after encountering social exclusion compared with encountering social inclusion. However, if the brand of the shop is well known, no significant difference exists in online reviews given by customers between social exclusion and social inclusion conditions.

### Theoretical Contributions

First, this study further expands the literature on the effect of offline behavior on online response behavior, which examines the effect of offline social exclusion on online reviews. Previous research has mostly focused on the Internet use behaviors, such as using the Internet or social media after individuals are socially excluded. For example, compared with those who have not experienced social exclusion, when individuals encounter social exclusion, those with high social anxiety prefer to use SNS. After manipulating social exclusion on participants, Knausenberger and Echterhoff (2018) found that participants could recover the threat of ownership brought by social exclusion through the use of SNS such as Facebook. The study of Lee and Chiou (2013) also showed that when under a social exclusion condition, after the inclusion motivation was aroused, individuals spent longer time addressing the font color used in SNS. When adolescents encounter social exclusion (vs. not encounter), online communication with a stranger of the opposite sex (vs. playing online games) can help them recover from the negative effect of social exclusion because online communication can supplement the self-esteem of young people and help them perceive the value of a relationship (Gross, 2006). According to the research results of Tom Tong and Walther (2011), new media (i.e., computational media communication) provides daters with new ways to express their love and refusal, mainly including, (1) one-click rejection and (2) no response. Moreover, the results of Chen (2012) showed that being rejected online (such as being rejected in SNS) will lead to negative emotions in self-reports and revenge on the site and the rejection group. Furthermore, men have a stronger desire for revenge than women. The results of Ahn and Shin (2013) showed that the use of social media not only can help people to avoid social loneliness but also help them seek associations. In terms of avoiding social loneliness, social media can replace face-to-face communication, whereas in terms of seeking associations, the use of social media can enhance face-to-face communication. The above studies suggested that social exclusion affects the Internet use behavior, especially in the recovery process of psychological threats brought by social exclusion. However, these studies did not consider the influence of social exclusion on online reviews. Therefore, the present study focused on the influence of offline social exclusion on online reviews, which enriches the existing research.

Second, the present study further expands the mediating mechanism on the effect of offline social exclusion on the online behavior of an individual. As the existing studies have mostly used the recovery of psychological threat as the mediating mechanism, the present work found that situational attribution plays a mediating effect between offline social exclusion and individual behavior. Previous studies have suggested that social exclusion affects individual behavior mainly through four major belonging needs, namely, attribution needs, self-esteem need, control need, and the need for a meaningful existence (Williams et al., 2000). The extant research has mostly used one of the four belonging needs as the mediating mechanism for the influence of social exclusion on individual behavior. For example, Ward and Dahl (2014) argued that attribution needs explain how social exclusion leads to the preference of consumers for products from luxury brands. Duclos et al. (2013) found that self-esteem needs explain how social exclusion leads to individual preference to risky financial investment. Jaehoon and Shrum (2012) found that control needs can explain how social exclusion leads to individual show-off consumption behavior. Different from these studies, on the basis of situational attribution and self-serving bias theories, this study found that situational attribution may explain the influence of social exclusion on online reviews. As consumers encounter social exclusion, they attribute their unfortunate experience to the consumption scenarios, which leads to negative online comments.

Finally, this study enriches the literature on the antecedent variables on online reviews by taking offline social exclusion as one important factor. Numerous studies have investigated the antecedent variables on online reviews. For example, Ghazi (2017) found that the motives for writing positive and negative online reviews are different. Liu et al. (2019) proposed that those who have already written several reviews will pay more effort to pursue the reward from the company, and a large number of online reviews will eager others to participate in this process. Moses et al. (2018) examined the effect of different self-construal on participating in online reviews and designed an interactive online feature to figure out the social motives for writing reviews. However, to the best of our knowledge, no research takes offline social exclusion as the antecedent variable on online reviews, whereas offline social exclusion is an overly common scene in the offline consumption environment when individuals use online platforms to book something and consume offline.

### Practical Contributions

The two practical implications of the present study are as follows.

On the one hand, offline consumption experience often influences online reviews, in which, the present study finds that offline social exclusion leads to negative online reviews. As mention before, the example at the beginning of this study, being rejected by a woman in the restaurant may only lead to a negative online review of the man, as he attributes the unfortunate experience to the consumption scenario. To avoid such negative online reviews, storekeepers need to be careful about their services and products when serving consumers offline, such as the attitude of the waiter or waitress and responding speed to the needs of customers. By improving product and service quality, storekeepers can reduce the probability of customers attributing the failure of life of consumers to the store.

On the other hand, the present research finds that the brand awareness of stores can offset the effect of offline social exclusion on online reviews. Therefore, offline and online shops, especially those who are booked online and consumed offline, should improve their brand awareness through various methods, such as advertising and word-of-mouth marketing, which can reduce negative online reviews caused by the personal experience of consumers.

### Limitation and Future Research

There still exist some limitations in the present study. First, this study uses a fictional scenario to manipulate offline social exclusion, which may not reflect the actual consumption experience in reality. A short-time interval exists between scenario manipulation and measurement in the laboratory. In reality, after offline consumption, it may take some time to give online reviews on the online platform. Therefore, future research can use another method to manipulate the offline scenario or control the time interval, which may lead to a different result.

Second, in this study, most of the participants are university students, which indicates that the hypothesis can be verified adequately, but it still needs more participants from a different culture to be tested in these hypotheses. Under different cultural backgrounds, individuals may have different feelings toward relationship orientation. For example, consumers with different cultural tendencies have different reactions to brand scandals (Wei et al., 2013; Liang et al., 2018), which can be an important factor that influences the results in this study. Future research can exploit this factor to further test our hypothesis.

Finally, there may exist another mediating mechanism in the present study, which can explain the influence of offline social exclusion on online reviews. On the basis of self-protection and self-serving bias, this study proposes that situational attribution plays a mediating mechanism in the effect of offline social exclusion on online reviews. However, there may exist other mediating mechanisms to explain this effect, such as cognitive focus (Liang et al., 2021), psychological reactance (Liang et al., 2021), and cognitive need demand (Dong et al., 2018). These mechanisms need to be further examined in future research.

## Data Availability Statement

The raw data supporting the conclusions of this article will be made available by the authors, without undue reservation.

## Ethics Statement

The studies involving human participants were reviewed and approved by the Ethics Committee of Guangxi University. The patients/participants provided their written informed consent to participate in this study.

## Author Contributions

SL, YWa, and YaC contributed to the conception of the manuscript and helped to perform the analysis with constructive discussions. YuC and ZZ performed the experiment. YWu and SL contributed significantly to analysis and manuscript preparation. YWa and ZZ performed the data analyses and wrote the manuscript. All authors contributed to the article and approved the submitted version.

## Conflict of Interest

YWa was employed by Hualan Design (Group) Co., Ltd. The remaining authors declare that the research was conducted in the absence of any commercial or financial relationships that could be construed as a potential conflict of interest.

## Publisher’s Note

All claims expressed in this article are solely those of the authors and do not necessarily represent those of their affiliated organizations, or those of the publisher, the editors and the reviewers. Any product that may be evaluated in this article, or claim that may be made by its manufacturer, is not guaranteed or endorsed by the publisher.
