# Supplementary material for: “Scapegoat” for Offline Consumption: Online Review Response to Social Exclusion
Source: Front Psychol. 2021 Dec 2;12:783483. doi: 10.3389/fpsyg.2021.783483 (PMC8674422; doi:10.3389/fpsyg.2021.783483)
Supplement: Supplementary file 1 [file Presentation_1.pdf]

APPENDIX 1 READING MATERIALS FOR STUDY 1

Participants in group 1 (*peer and social exclusion*) are told to read the material as follows:

"If you were single and you were introduced to a friend of the opposite sex. You had developed a preoccupation with him or her and were eager to have dinner alone with him or her to get to know each other better. Just then you found that he (she)was free so you invited him (her) to have a meal together, and he (she) also agreed. You found a special restaurant through Dianping.com and book a set meal for two from this restaurant on Dianping.com. Then you joined your date at the restaurant at the appointed time. During the meal, he or she showed dissatisfaction to you because he or she thought you apperance are so bad, and after the meal, he or she refused to continue the relationship. When you got home, Dianping.com prompted you with a message to rate the restaurant."

Participants in group 2 (*peer and social inclusion*) are told to read the material as follows:

"If you were single and you were introduced to a friend of the opposite sex. You had developed a preoccupation with him or her and were eager to have dinner alone with him or her to get to know each other better. Just then you found that he (she)was free so you invited him (her) to have a meal together, and he (she) also agreed. You found a special restaurant through Dianping.com and book a set meal for two from this restaurant on Dianping.com. Then you joined your date at the restaurant at the

appointed time. During the meal, he or she showed approval to you because he or she thought you apperance are so good, and after the meal, he or she made clear that he or she wanted to continue the relationship. When you got home, Dianping.com prompted you with a message to rate the restaurant."

Participants in group 3 (*waiter and social exclusion*) are told to read the material as follows:

"Suppose you invited a friend to have dinner together. You found a special restaurant through Dianping.com and booked a set meal for two from this restaurant on Dianping.com. Then you joined your friend at the restaurant at the appointed time. During the meal, the waiter showed dissatisfaction to you, and after the meal, the waiter clearly expressed dissatisfaction with your low consumption behavior. When you got home, dianping.com prompted you with a message to rate the restaurant."

Participants in group 4 (*waiter and social inclusion*) are told to read the material as follows:

"Suppose you invited a friend to have dinner together. You found a special restaurant through Dianping.com and booked a set meal for two from this restaurant on Dianping.com. Then you joined your friend at the restaurant at the appointed time. During the meal, the waiter showed various hospitality to you, and after the meal, the waiter clearly expressed appreciation to you with your generous consumption behavior. When you got home, dianping.com prompted you with a message to rate the restaurant."
