# Supplementary material for: “Scapegoat” for Offline Consumption: Online Review Response to Social Exclusion
Source: Front Psychol. 2021 Dec 2;12:783483. doi: 10.3389/fpsyg.2021.783483 (PMC8674422; doi:10.3389/fpsyg.2021.783483)
Supplement: Supplementary file 2 [file Presentation_2.pdf]

APPENDIX 2 READING MATERIALS FOR STUDY 2

The participants in group 1 (*social exclusion and well-known*) are told to read the following material:

"If you were single and you were introduced to a friend of the opposite sex. You had developed a preoccupation with him/her and were eager to have dinner alone with him/her to get to know each other better. Just then you found that he/she was free so you invited him/her to have a meal together, and he/she also agreed. You found Haidilao hot pot restaurant through Dianping.com and booked a set meal for two from this restaurant on Dianping.com. Then, you joined your date at the Haidilao restaurant at the appointed time. During the meal, he/she showed dissatisfaction to you because he/she thought that you looked bad, and after the meal, he/she refused to continue the relationship. When you got home, Dianping.com prompted you with a message to rate the restaurant."

The participants in Group 2 (*social exclusion and less-known*) are told to read the following material:

"If you were single and you were introduced to a friend of the opposite sex. You had developed a preoccupation with him/her and were eager to have dinner alone with him/her to get to know each other better. Just then you found that he/she was free so you invited him/her to have a meal together, and he/she also agreed. You found Xiaofuzi hot pot restaurant through Dianping.com and booked a set meal for two from this restaurant on Dianping.com. Then, you joined your date at the Xiaofuzi restaurant

at the appointed time. During the meal, he/she showed dissatisfaction to you because he/she thought that you looked bad, and after the meal, he/she refused to continue the relationship. When you got home, Dianping.com prompted you with a message to rate the restaurant.”

The participants in Group 3 (*social inclusion and well-known*) are told to read the following material:

“If you were single and you were introduced to a friend of the opposite sex. You had developed a preoccupation with him/her and were eager to have dinner alone with him/her to get to know each other better. Just then you found that he/she was free so you invited him/her to have a meal together, and he/she also agreed. You found Haidilao hot pot restaurant through Dianping.com and booked a set meal for two from this restaurant on Dianping.com. Then, you joined your date at the Haidilao restaurant at the appointed time. During the meal, he/she showed approval to you because he/she thought that you looked good, and after the meal, he/she made clear that he/she wanted to continue the relationship. When you got home, Dianping.com prompted you with a message to rate the restaurant.”

The participants in Group 4 (*social inclusion and less-known*) are told to read the following material:

“If you were single and you were introduced to a friend of the opposite sex. You had developed a preoccupation with him/her and were eager to have dinner alone with him/her to get to know each other better. Just then you found that he/she was free so

you invited him/her to have a meal together, and he/she also agreed. You found Xiaofuzi hot pot restaurant through Dianping.com and booked a set meal for two from this restaurant on Dianping.com. Then, you joined your date at the Xiaofuzi restaurant at the appointed time. During the meal, he/she showed approval to you because he/she thought that you looked good, and after the meal, he/she made clear that he/she wanted to continue the relationship. When you got home, Dianping.com prompted you with a message to rate the restaurant.”
